# Fever, Haematuria, and Acute Graft Dysfunction in Renal Transplant Recipients Secondary to Adenovirus Infection: Two Case Reports

**DOI:** 10.1155/2013/195753

**Published:** 2013-02-14

**Authors:** J. Ramírez, I. C. Bostock, A. Martin-Onraët, S. Calleja, A. Sánchez-Cedillo, L. A. Navarro-Vargas, A. L. Noriega-Salas, O. Martínez-Mijangos, N. O. Uribe-Uribe, M. Vilatoba, B. Gabilondo, L. E. Morales-Buenrostro, J. Alberú

**Affiliations:** ^1^Department of Transplantation, National Institute of Medical Sciences and Nutrition, 14000 Mexico City, DF, Mexico; ^2^Department of Infectious Disease, National Institute of Medical Sciences and Nutrition, 14000 Mexico City, DF, Mexico; ^3^Department of Anatomic Pathology, National Institute of Medical Sciences and Nutrition, 14000 Mexico City, DF, Mexico; ^4^Department of Nephrology-Mineral Metabolism, National Institute of Medical Sciences and Nutrition, 14000 Mexico City, DF, Mexico

## Abstract

We report two cases of adenoviral infection in kidney transplant recipients that presented with different clinical characteristics under similar demographic and posttransplant conditions. The first case presented with fever, gross haematuria, and acute graft dysfunction 15 days following renal transplantation. A graft biopsy, analyzed with immunohistochemistry, yielded negative results. However, the diagnosis was confirmed with blood and urine real-time PCR for adenovirus 3 days after the initial clinical manifestations. The immunosuppression dose was reduced, and ribavirin treatment was started, for which the patient quickly developed toxicity. Antiviral treatment allowed for transient response; however, a relapse occurred. The viral real-time PCR became negative upon immunosuppression reduction and administration of IVIG; graft function normalized. In the second case, the patient presented with fever and dysuria 1 month after transplantation. The initial imaging studies revealed graft enlargement and areas of hypoperfusion. In this case, the diagnosis was also confirmed with blood and urine real-time PCR for adenovirus 3 days after the initial clinical manifestations. Adenoviral nephritis was confirmed through a graft biopsy analyzed with light microscopy, immunohistochemistry, and PCR in frozen tissue. The immunosuppression dose was reduced, and IVIG was administered obtaining excellent clinical results along with a negative real-time PCR.

## 1. Introduction

In the scenario of transplantation, infection with adenovirus is more common after bone marrow transplantation and affects children more commonly than adults (31–47% versus 13.6%) with an estimated mortality of 26% in symptomatic patients [1, 2].

Adenovirus infection, particularly with serotypes B 7, 11, 34, and 35, presents more frequently as hemorrhagic cystitis and is usually a rare and limited infection in renal transplant recipients [3]. A disseminated disease with more serious systemic complications or death is uncommon (58% of cases are self-limited). Throughout the literature there have only been a small number of case reports of adenoviral-related nephritis in kidney transplant recipients [2–10]. In this report, we describe two unusual cases of early onset infection with adenovirus, as well as a review of previous cases reported in the literature.

## 2. Case 1

A 26-year-old man with end-stage renal disease of undetermined etiology received a living donor renal transplantation on February 2012. The adult donor was his sister sharing 1-haplotype; the AHG-CDC cross match was negative, the PRA result was 1% and 0% for class I and class II, respectively. Time zero graft biopsy was not performed due to a limited subcapsular hematoma in the superior pole. He received basiliximab induction, followed by methylprednisolone, tacrolimus (TAC), and mycophenolate mofetil (MMF). Trimethoprim-sulfamethoxazole was given for bacterial prophylaxis. He was determined to have a low-risk CMV (D−/R−) status so no viral prophylaxis was administered in this regard. On day 5-posttransplantation (post-KT), a Doppler US was performed in which adequate renal perfusion and normal graft characteristics were observed. He was discharged on day 6 post-KT with a serum creatinine (SCr) of 1.2 mg/dL and an immunosuppressive regimen based on TAC, MMF, and prednisone administered at a dose of 6 mg/BID (trough levels 8 ng/dL), 500 mg/BID, and 10 mg/day, respectively. 

After an uneventful posttransplant evolution, the patient was hospitalized 9 days later due to gross haematuria, dysuria, and an elevation of SCr to 1.5 mg/dL. A complete workup was performed. CBC showed Hb 11 g/dL, leukocytes 5.8 K/uL, and platelets 238 K/uL. Urine analysis showed 49 leukocytes and 135 erythrocytes per each high-power field. Ceftriaxone 2 g IV BID was administered for a possible urinary tract infection (UTI) along with IV fluids. His vital signs were as the following: temperature 36.7°C, blood pressure 130/65 mm/Hg, pulse rate 100 bpm, and respiratory frequency 20/min. 

**Figure 1:**
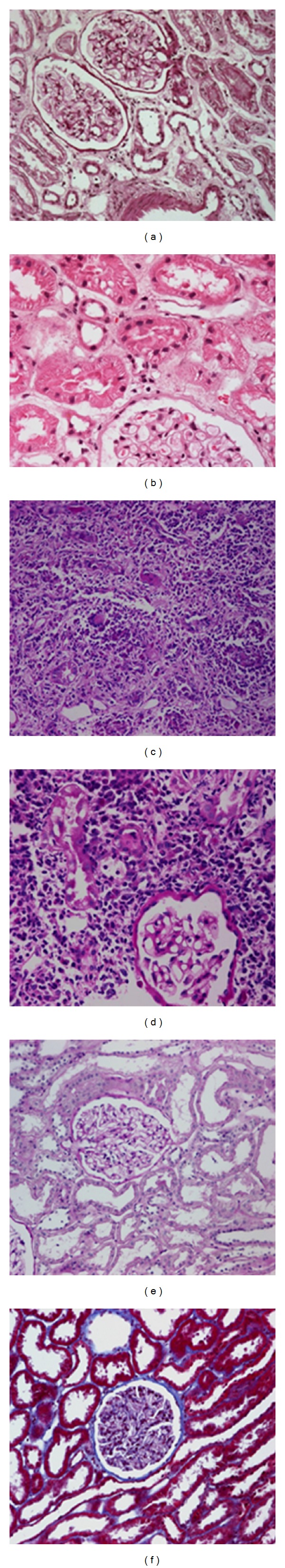
Renal graft biopsies. In case 1, histological findings were irrelevant; no viral inclusions were identified by light microscopy (H&E (a) and (b)) or immunohistochemistry. Renal biopsy of case 2 demonstrated focal interstitial necrotizing granulomata with neutrophils, plasma cells, and lymphocytes ((c) H&E and (d) PAS stain); note that the glomeruli are not affected. Control graft biopsy only demonstrated minimal interstitial fibrosis ((e) PAS stain and (f) Masson trichromic stain).

On day 1 of hospitalization, the patient developed fever of 39°C which was treated with paracetamol. Leukopenia, anemia, and thrombocytopenia became evident with Hb 7.5 mg/dL, leukocyte 2.0 K/uL, and platelets 124 K/uL. There was also an increase in SCr to 2.04 mg/dL. A Doppler US was performed in which an image compatible with a renal graft residual hematoma, evidence of moderate hydronephrosis and blood clots in the bladder, were observed. Computed tomography (CT) scan studies of the chest, abdomen, and pelvis exclusively showed a residual fluid collection surrounding the graft. Urine and blood cultures were negative for bacteria. A graft biopsy was performed which showed normal histology on light microscopy; routine panel of immunofluorescence was negative. Immunohistochemistry for adenovirus (cell marque 1 : 100), CMV (cell marque 1 : 50), SV-40 (cell marque 1 : 100), and LMP1 (cell marque 1 : 100) did not demonstrate the presence of viral particles (Figures 1(a) and 1(b)). Cytomegalovirus (CMV) pp65 antigenemia and parvovirus B19 antigenemia were negative. BK virus was also negative in blood and urine analysis. On day 3, blood and urine real-time qualitative polymerase chain reaction for adenovirus yielded positive results. 

On day 4 of hospitalization, a cystoscopy was performed due to persistent haematuria. There was evidence of clots but no active bleeding, although abnormal mucosal inflammation and petechia were evident. MMF was withdrawn, and the dose of TAC was reduced to reach a blood level of 5 ng/dL (previous blood level 15.3 ng/mL). The patient continued to be febrile with a temperature of 39°C, for which ceftriaxone was substituted by 1 g IV ertapenem QD. Twenty-four hours later, ertapenem was replaced by meropenem. A continuous cystoclysis was placed through the Foley catheter to relieve the haematuria. There was a SCr decrease to 1.53 mg/dL.

On day 7 of hospitalization, ribavirin was started at a dose of 800 mg/6 hr. It is worth mentioning that the antiviral drug was not available before this day. Renal function showed further deterioration with an elevation of SCr to 1.95 mg/dL, leukocytes count was 4.2 K/uL, platelets 233 K/uL, and Hb was 7.9 mg/dL. The fever persisted with a temperature of 40°C. On day 9, the SCr decreased to 1.48 mg/dL. 

The treatment for adenovirus continued with ribavirin 800 mg/6 hr for 10 days until the dosage was reduced to 400 mg/6 hr due to evidence of leukopenia. The treatment continued for a total of 20 days until the urine and blood real-time PCR was negative and the patient was asymptomatic; the urine analysis was normal as well. Fifteen days later, the patient returned with gross haematuria, fever, and a positive blood real-time PCR for adenovirus. He received treatment with IVIG with a total dose of 2 g/kg. He was later discharged with a stable renal function.

**Figure 2:**
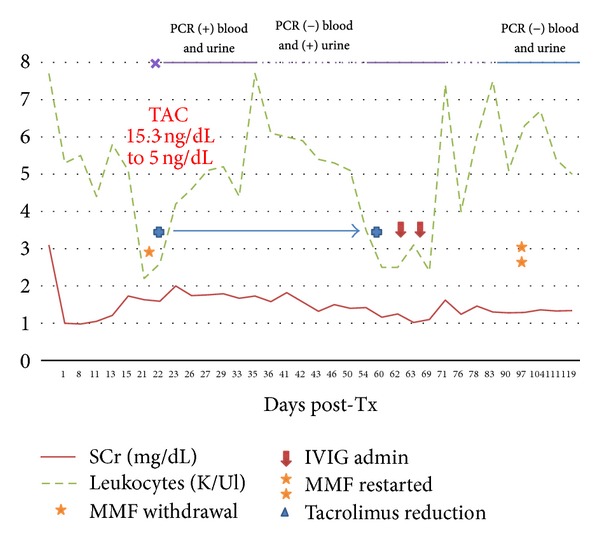
Case 1: correlation of SCr, leukocytes, and treatment administration.

Eight months later, his renal function is stable with aSCr of 1.33 mg/dL and is currently on treatment with TAC 10 mg/day (8.5 ng/dL), MMF 500 mg/day, and prednisone 5 mg/day. Viral serology for CMV with pp65 antigenemia and parvovirus B19 antigenemia has remained negative. Blood and urine real-time PCR for adenovirus has remained negative in 3 serial subsequent determinations (Figure 2). A subsequent control graft biopsy was not performed. 

## 3. Case 2

A 27-year-old man with end-stage renal disease secondary to systemic lupus erythematosus underwent renal transplantation on June 2012. The adult donor was his brother sharing 2-haplotypes, the AHG-CDC cross match was negative, and the PRA result was 0% and 0% for class I and class II, respectively. He was determined to have an intermediate CMV risk status (D+/R+). Time zero graft biopsy was performed without significant alterations. Trimethoprim-sulfamethoxazole was given for bacterial prophylaxis. He received induction with basiliximab and triple drug therapy with TAC, MMF, and prednisone. On day 5 posttransplantation (post-KT), a Doppler US was performed in which adequate renal perfusion and normal graft characteristics were observed. He was discharged on day 6 post-KT with a SCr of 1.3 mg/dL and an immunosuppressive regimen based on TAC, MMF, and prednisone administered at a total dose of 5 mg/BID (trough levels 9.1 ng/dL), 1 g/BID, and 10 mg/day, respectively.

After an uneventful posttransplant evolution, the patient was admitted to the emergency department 1 month later due to constant fever of 38°C and severe dysuria. During his initial evaluation, he had stable vital signs without any apparent alteration on physical examination. His SCr showed an elevation to 1.6 mg/dL. A complete workup was performed. CBC showed Hb of 13.9 g/dL, leukocytes 5.7 K/uL, and platelets 265 K/uL. Urine analysis showed 6 leukocytes, 0 erythrocytes, and 4 bacteria per each high-power field.

A graft ultrasound revealed and increment of size and hypoechogenicity of the graft and data suggesting parenchymal hypoperfusion; however, arterial and venous blood flow were preserved. An abdominal CT scan corroborated these acute changes and showed evidence of perirenal fat inflammation and ureteral prominence. Treatment with ertapenem IV was begun.

**Figure 3:**
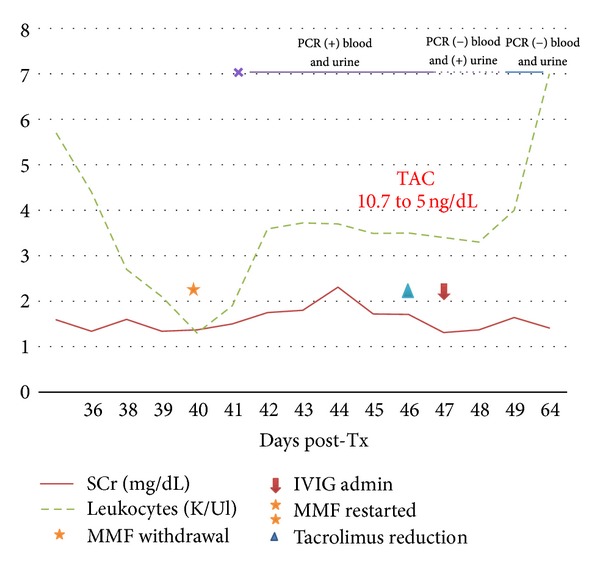
Case 2: correlation of SCr, leukocytes, and treatment administration.

On day three of hospitalization, the SCr was 1.75 mg/dL. Both urine and blood bacterial cultures yielded negative results; cytomegalovirus (CMV) pp65 antigenemia and parvovirus B19 antigenemia were negative. BK virus was also negative in blood and urine analysis. Analysis by real-time PCR of blood and urine were positive for adenovirus. A graft biopsy was also performed revealing histopathological findings consistent with adenovirus nephritis (Figures 1(c) and 1(d)). Viral particles or inclusions were not demonstrated by electron microscopy or immunohistochemistry, respectively. Routine immunofluorescence panel was negative; the remaining frozen tissue was positive for adenovirus real-time PCR. The immunosuppressive regimen was modified by MMF withdrawal and tapering the dose of TAC to reach a serum blood level of 5 ng/mL. The dose of prednisone was maintained at 5 mg/day. On the fourth day of hospitalization, IVIG was administered at a total dose of 1 g/kg. The antibiotic treatment was suspended due to persistently negative blood and urine bacterial cultures. The decision to administer IVIG along with reducing the immunosuppression was made in order to elucidate if this treatment modality would be efficient in clearing the viral infection. After the administration of IVIG, a reduction in the SCr to 1.31 mg/dL was observed, correlating with an improvement in the patient's symptoms and the absence of fever. The patient was discharged 10 days later showing an adequate response to treatment although the SCr showed a recurrent elevation stabilizing at a new level of 1.64 mg/dL. Blood and urine real-time PCR for adenovirus were negative on two subsequent determinations (Figure 3). Posterior SCr determination decreased to 1.4 mg/dL, and a control graft biopsy performed 2 months after discharge showed less than 5% of interstitial fibrosis, without inflammatory infiltrate or other acute change (Figures 1(e) and 1(f)).

A comparison of the clinical presentation, laboratory results, and treatment modalities between both cases is depicted in Table 1. 

## 4. Discussion

There have been very few cases of adenovirus nephritis in renal transplant recipients reported in the literature. Kolankiewicz et al. published a case report and a review of the literature in which they analyzed 11 cases of renal transplant-related adenoviral nephritis proven with graft biopsy analysis and subsequent immunohistochemistry reported since 1998 [4–9, 11–13]. Two were children and the others were adults with a female to male ratio of 7 : 4. Most of the cases (8/11) presented within 8 months of the transplant, at a mean of 2.9 months with a range of 1–8 months. Three cases presented later, at 17, 18 and, 144 months, respectively. In this review, the patients commonly presented with gross haematuria and dysuria (10/11), fever (9/11), and acute renal failure (9/11) [10], and 27% of the patients had significant graft function impairment after adenoviral nephritis [10].

In kidney transplant recipients who present with fever, haematuria, acute kidney injury, and obstructive uropathy, adenoviral nephritis should be considered. The mean time of presentation reported is 3 (1–8) months, as were the cases reported herein, although late onset disease (17 and 144 months) causing obstructive uropathy has been reported as well [10]. 

Based on the clinical characteristics of the cases detailed in this review, it is evident that the clinical presentation in a case of adenovirus nephritis can be associated with variable clinical manifestations. Fever and an elevation in SCr can be the only manifestation of adenovirus infection, and the extent of the initial symptomatology does not correlate with the establishment of irreversible renal damage a posteriori [10]. How can we sustain “adenoviral nephritis” in the first case where no histological virus-related nephritis was found? Possibly it will be necessary to mention that even though no histological confirmation existed in the first case, the blood and urine real-time PCR + along with cystitis make the diagnosis likely. 

Although *in situ* hybridization of adenovirus in paraffin-embedded tissue should be the gold standard for diagnosis and treatment guidance, it is important to consider the clinical characteristics of the patient as the basis for clinical decisions. Kolankiewicz et al. reported in their series that 9 out of 11 cases had granulomatous changes, and 8 out of 11 had necrotizing inflammation, while 1 case had interstitial nephritis only, without necrotizing changes or granulomas. In this report, case 1 had a normal graft biopsy, while case 2 presented focal interstitial nephritis with necrotizing granulomata typical of adenovirus nephritis. 

In case 2, even though the degree of inflammatory damage in the graft secondary to adenoviral nephritis was expected to result in evident interstitial fibrosis, <2% was found in the followup graft biopsy performed 2 months after the original diagnosis. We must recall that this is a focal event, and possibly scarred areas were not sampled leading to a possible sampling bias. It is also important to highlight that in both cases, the SCr showed a tendency to decrease to basal levels, although in case 2 the extent of initial renal damage seemed to be more evident. 

Although the diagnosis of adenoviral infections classically depends on histology and viral culture, and biopsy remains the gold standard, molecular techniques have become more attractive due to increased sensitivity and rapid results with noninvasive techniques [14]. Sensitivity of nucleic acid amplification relies on the primers used, specimen detection and the characteristics of the method used. In these 2 cases, DNA detection was done by a multiplexed, real-time PCR, using 5 primers which allowed the detection of a wide range of types of adenovirus including subgroups A to C [15]. 

According to the definition of infection, case 2 should be considered as a definite case, since confirmation was done with histologic consistent findings, in addition to viremia, viruria and symptoms. In case 1, a possible adenoviral infection diagnosis was made by viremia, viruria, and consistent symptoms [14]. Many studies have shown that viremia can predict invasive disease and can be used for followup, even as a prognostic marker. Controversy remains for its interpretation in asymptomatic patients since results have been contradictory, but this was not the scenario of these 2 patients. In both cases the reversal of viremia and viruria correlated with resolution of symptoms. Even if this real-time PCR was not quantitative, it was useful to correlate symptom improvement with the absence of viremia.

The first therapeutic approach should be the reduction of immunosuppression allowing the immune system to mount a response against the virus and content viral replication. Concomitant intravenous immunoglobulin, cidofovir, and ribavirin have been reported as successful adjunctive therapies [14]. The addition of IVIG seemed to be a good alternative to antiviral medication, particularly in the scenario of drug toxicity or leukopenia. It is difficult to speculate whether the sole reduction of immunosuppression would have been enough to control adenoviral infection in the cases herein presented. The severity of the symptoms in both of them along with the graft dysfunction documented prompted us to give some adjunctive treatment modality. It seems that the combination of immunosuppressive drug reduction and IVIG were effective to control the adenoviral infection. In further detail, it is important to mention that ribavirin as a treatment for infection with adenovirus is the most effective against the serotype subgroup C (most cases of adenoviral nephritis are caused by serotype subgroup B) and has not shown to be clinically effective in previous episodes reported in the literature, although some authors still recommend it [3, 14]. In the cases report herein presented, the effect of ribavirin on case 1 can be considered only partially efficacious since the viral load disappeared after treatment but became positive in blood real-time PCR 15 days later, translating into an insufficient host response and antiviral effect against the infection.

In synthesis, when this diagnosis is confirmed, the most important therapeutic intervention is the reduction of immunosuppression, although additional antiviral therapy may be beneficial [14]. In patients with a more severe clinical presentation, reduced immunosuppression and concomitant cidofovir, ribavirin and IVIG have been successfully employed [14]. The role of IVIG has also been described to be useful in the treatment and prevention of posttransplant infectious complications including CMV, parvovirus B19 and polyoma BK virus [16]. 

The diagnosis and treatment of an infection with adenovirus should follow a quick and effective process in order to prioritize renal graft function preservation and achieve an adequate host response. The reduction of immunosuppression along with the administration of IVIG seems to provide a favorable outcome in this scenario.

## Figures and Tables

**Table 1:** Clinical manifestations and treatment response.

Case 1	Case 2
1-Haplotype-match	2-Haplotypes match
Induction therapy with Basiliximab	No induction therapy
21 days posttransplantation	1 month posttransplantation
Gross hematuria and dysuria	Severe dysuria
No fever (36.7°C)	Fever (38°C)
Urine analysis with 49 leukocytes and 135 erythrocytes per each high-power field.	Urine analysis with 6 leukocytes and 4 bacteria per each high-power field.
Hb 11 g/dL, leukocytes 5.8 K/uL, and platelets 238 K/uL.	Hb 13.9 g/dL, leukocytes 5.7 K/uL, and platelets 265 K/uL.
Renal ultrasound with moderate hydronephrosis, normal blood flow.	Renal ultrasound with increase in size and echogenicity, data suggesting hypoperfusion.
Normal graft biopsy	Graft biopsy with histopathological changes and (+) real-time PCR for adenovirus
Blood and urine real-time PCR (+) for adenovirus; CMV and BK virus serology (−)	Blood and urine real-time PCR (+) for adenovirus; CMV and BK virus serology (−)
TAC dose reduced, MMF suspended	TAC dose reduced, MMF suspended
Treatment with ribavirin and IVIG	Treatment with IVIG only
SCr returned to basal level (1.3 mg/dL) after treatment.	SCr remained elevated (1.6 mg/dL) but with a recent tendency to decrease (1.4 mg/dL). Control graft biopsy with <2% fibrosis. Control graft biopsy with <2% fibrosis.
